# It is time for open access in clinical care

**DOI:** 10.7554/eLife.77184

**Published:** 2022-01-28

**Authors:** Edy Kim

**Affiliations:** 1 Division of Pulmonary and Critical Care Medicine, Department of Medicine, Brigham and Women's Hospital Boston United States; 2 Harvard Medical School Boston United States

**Keywords:** sparks of change, research culture, COVID-19, medicine, open access, clinical guidelines

## Abstract

A healthcare center widely sharing its internal guidelines on how to treat COVID-19 patients “just wasn’t done.” As the pandemic raged at a Boston hospital, the next generation of clinical leaders pushed for change.

It’s the early days of March 2020, and things are starting to fall into place for me. After many years training as a physician-scientist, I’m setting up my own lab at the Brigham and Women’s Hospital in Boston, Massachusetts. Most of my week is protected for my research on pulmonary and critical care medicine, with only 20% of my time spent with patients in intensive care. Still, the unit director has just asked me to attend a few meetings on COVID-19, “as a backup.”

A few weeks later, an entire hospital building has been turned into a COVID-19 intensive care unit. I have stopped doing my research, and I don’t even have time to check with my lab members about their own progress. My pager keeps going off: yet another patient has been intubated and needs a bed in intensive care. Clinicians in specialty clinics are pulled to be COVID-19 interns. We make so many color-coded teams that we run out of options and a “Teal team” is dangerously close on the horizon. A meal is protein bars and I sleep on the couch next to my desk – every minute counts.

Every day we update anxious families as their loved ones struggle in our unit. One morning I try but fail to stop a patient’s lung from filling with blood. There is nothing more we can do. His wife is angry – at us, but really at this new world we’ve been thrown in. His daughter keeps thanking us. It only makes us feel worse.

How should we care for these patients? What kind of treatments could we use, for whom and when? We had no guidelines for COVID-19. In normal times, standards of care are developed over decades. Experts gather every few years to assess peer-reviewed, randomized controlled trials and make incremental changes that are published months later in a journal; the typical physician catches up at the next year’s conference. But there was nothing normal about a pandemic growing exponentially, and every week mattered.

As cases were ramping up in March 2020, I had started to gather experts from across our medical center so we could analyze emerging evidence and put together our hospital’s treatment guidelines on the fly. Data were scarce initially. We started with phone calls to colleagues in Italy or China, then case reports; later, we even faced the dilemma of recommending the drug dexamethasone based on a press release from the clinical trial.

We could not wait months for peer-reviewed publications, so we relied heavily on preprints. Critically evaluating those articles then became our primary challenge, and I was grateful for my knowledge of inflammation and my ability to assess papers (those journal clubs in grad school were paying off!). Being a physician-scientist can sometimes feel like juggling two jobs badly, but now I was helping to bridge science and the bedside. For example, we began to use the monoclonal antibody tocilizumab based on our review of preprints, even though targeted immunotherapy had never previously been standard of care for acute respiratory distress syndrome.

Around mid-March, our hospital had put together its first internal guidelines to treat COVID-19 patients. We were about to upload them as a PDF to our hospital’s internal server for our staff to download and print them, when the next generation of clinical leaders spoke up. As pulmonary fellow-in-training Dr C Lee Cohen pointed out, how could paper printouts possibly keep up with the rapidly evolving data on COVID-19? Just as importantly, she and others suggested that we had a responsibility to share our findings with the world, not just with our staff. In anticipation, Cohen built the website COVIDprotocols.org to host our guidelines: this platform was web and mobile-based, searchable and could be continually updated.

Evolving recommendations, accessible on our smartphones? That idea was an instant hit. Sharing them outside our hospital was not. What if we were wrong and misled professionals around the globe? Making internal guidelines widely public *just wasn’t done*. I hesitated. As the individual who had put together the interdisciplinary team working on our COVID-19 protocols, I felt ultimately responsible for any negative fallout. But the pandemic was too massive, and the global confusion too overwhelming; it wasn’t perfect, but it just had to be done. Despite our doubts, on March 20, 2020, we launched the website Cohen had built, and released our first set of guidelines to the public.

**Figure 1:**
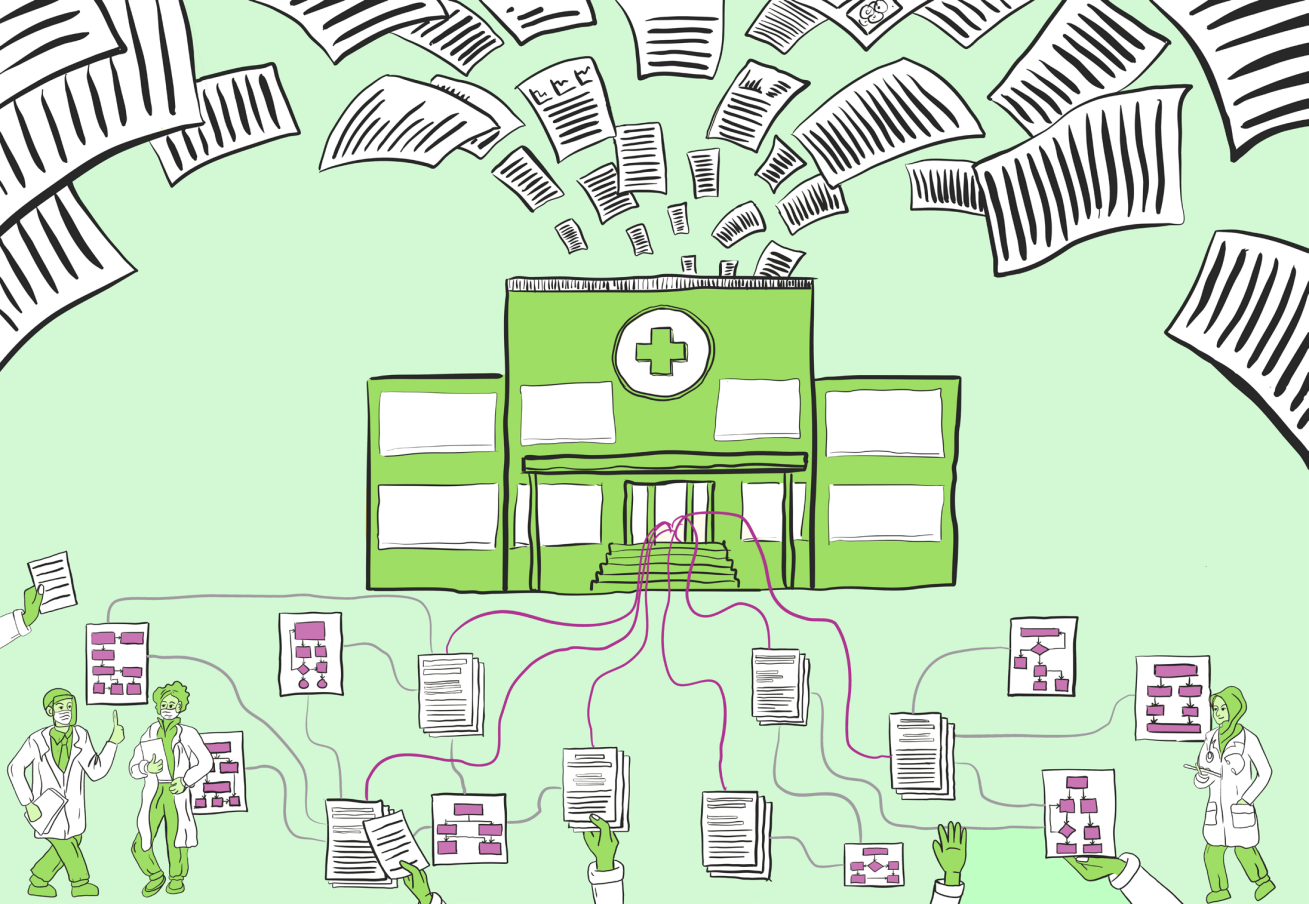
Poring over preprints and emerging data, a team of physicians at the Brigham and Women’s Hospital in Boston put together guidelines on how to treat COVID-19 patients. These were then shared openly in the early days of the pandemic.

IMAGE CREDIT: Vivid Biology (@vividbiology; http://www.vividbiology.com/)

Within days we heard from healthcare staff around the world. “Thank you, thank you, thank you”, wrote the director of the ICU at a teaching hospital in Cambridge, Massachusetts: “When I saw [the website] – I gave a huge sigh of relief. It will help the tiny places who don’t have the bandwidth or manpower to do this on their own.” My own brother-in-law, a hospitalist physician, used it to help lead care at his community hospital, which only had a handful of intensive care physicians. Bursts of page views from new countries were often followed, a few weeks later, by newspapers reporting how COVID-19 was surging in these parts of the world.

In retrospect, it is hard to understand why it took so long for open access in clinical care to emerge. To date, the website has had over half a million users, coming from more than 190 countries. It covers the full range of clinical treatment, from diagnostic tests to critical care. Each section pairs a literature review with detailed protocols. For example, a physician could read the evidence for tocilizumab and then follow step-by-step guides for treating patients. Originally, COVIDprotocols.org was most useful for academic medical centers in resource-rich countries, but in 2021 it was updated to be relevant for healthcare teams with limited assets as well.

Nearly two years later, my hospital can now manage the waxing and waning streams of COVID-19 patients with normal capacity. My schedule has returned to meetings with scientific collaborators, I have just submitted my first major NIH grant applications, and we are finishing manuscripts that had to be put on hold. But my lab meetings remain over Zoom, and I still spend the occasional night in my office. Old habits die hard.

Not all of them though. Until COVID-19, I had been reluctant to post preprints or even present at conferences, unless it was to help grant submissions. I felt the world should see our most rigorous work, improved by the peer-review process. To be honest, I also wanted to avoid getting scooped. Whether a journal was open access never crossed my mind.

Now, I deeply feel that the default should be early sharing of data, before peer review. Many physicians and hospitals lack the luxury of unlimited access to scientific literature. Scientific journals making their COVID-19 articles open access and free to publish spurred on a global burst of scientific research that included places which otherwise would have been left aside. I hope that these conceptual shifts will outlive the global misery of the pandemic.

## Acknowledgements

I thank Dr. C. Lee Cohen for her leadership of COVIDprotocols.org while a pulmonary and critical care fellow at the Brigham and Women’s Hospital and Harvard Medical School. I thank Dr. L. T. “Chip” Merriam for comments on the manuscript. I thank the many nurses, respiratory therapists, pharmacists, dieticians, and physicians for their contributions to the COVID-19 guidelines and direct care of COVID-19 patients.

## Share Your Experiences

This article is a Sparks of Change column, where people around the world share moments that illustrate how research culture is or should be changing. Have an interesting story to tell? See what we’re looking for and the best ways to get in touch here.

